# Editorial: Manipulation of plant architecture for crop production

**DOI:** 10.3389/fpls.2024.1502833

**Published:** 2024-10-14

**Authors:** Zoe Hilioti, Dulce Antunes, Panagiotis Kalaitzis, Georgios Merkouropoulos

**Affiliations:** ^1^ Centre For Research and Technology Hellas (CERTH), Institute of Applied Biosciences, Thessaloniki, Greece; ^2^ University of Algarve, Mediterranean Institute for Agriculture, Environment and Development, Faro, Portugal; ^3^ Mediterranean Agronomic Institute of Chania, Chania, Greece; ^4^ Department of Vitis, Hellenic Agricultural Organization-DIMITRA (ELGO-DIMITRA), Institute of Olive Tree, Subtropical Crops and Viticulture, Athens, Greece

**Keywords:** plant architecture, crops, production, genetics, breeding

Plant architecture, encompassing the spatial arrangement and morphological characteristics of plant organs, plays a crucial role in agricultural productivity. This Research Topic presents current research and review on the manipulation of plant architecture in major crops, highlighting key findings from genome-wide association studies (GWAS), quantitative trait loci (QTL) mapping, Simple Sequence Repeat (SSR) markers, and genetic manipulation efforts.

In the context of agricultural productivity, plant architecture refers to the three-dimensional organization of various plant structures, including stems, leaves, and roots. This organization directly affects a plant’s ability to capture sunlight, utilize nutrients, and ultimately yield harvestable biomass. Recent advancements in molecular biology and genetics have provided new opportunities to manipulate these architectural traits for improved crop performance. The challenges presented by climate change, including altered precipitation patterns, increased pest pressures, and extreme temperatures, make this research particularly timely and relevant.

Rice (*Oryza sativa* L.) is a staple food for more than half of the world’s population, making its yield stability essential for food security. As climate change and environmental stresses become more prevalent, enhancing rice varieties with both ideal plant architecture and stress tolerance has emerged as a critical area of research. In this Research Topic, Zhao et al. provide a comprehensive review of the regulatory mechanisms linking plant architecture, stress tolerance, and biological defense, thus laying a foundational framework for the genetic networks that govern the synergistic improvement of these interrelated traits. This paper discusses the implications of their findings and how breeding initiatives can effectively incorporate these insights for improvement of multiple traits.

One of the critical limiting factors for rice productivity is lodging, an occurrence where the plant stems bend or break under stress from environmental factors or heavy grain loads. Lodging not only affects yield but also poses challenges for harvesting and crop management. Thus, understanding the genetic basis of lodging resistance, particularly through evaluating culm morphology and strength, is essential for plant breeding programs focused on increasing rice resilience and yield. Utilizing Genotyping by Sequencing (GBS) and a GWAS study involving 181 rice genotypes, Badri et al. identified significant genetic markers associated with culm strength in rice. Key findings include the detection of sub-groups within the genotypes, significant heritability for culm strength traits, and the identification of novel marker-trait associations (MTAs) with implications for breeding strategies.

In maize (*Zea mays* L.), plant architecture is essential for optimizing agricultural output and adaptability to various cultivation conditions. Two key traits within this architecture are plant height (PH) and ear height (EH). The ability of maize to achieve optimal photosynthetic efficiency is closely linked to these traits, as taller plants with higher ear placements can capture sunlight more effectively and support greater leaf area. Moreover, suitable PH and EH are critical for enhancing the crop’s tolerance to high plant densities and facilitating mechanical harvesting, which has become increasingly important in modern agricultural practices. Understanding the genetic basis underlying PH and EH can inform breeding strategies aimed at improving maize cultivars. Yang et al. investigated the QTL associated with these traits through rigorous mapping in recombinant inbred lines (RIL) and immortalized backcross (IB) populations, followed by candidate gene identification based on co-expression network analyses. They found significant QTLs influencing PH and EH in maize, providing a foundation for future genetic studies and breeding initiatives. The identification of candidate genes linked to these traits reinforces the potential for genetic manipulation to enhance agricultural productivity and adaptability.

Successful breeding strategies for improving agricultural yield increasingly depend on the utilization of genetic markers that aid in the selection of desirable traits. In *Brassica* species, notably Indian mustard (*Brassica juncea*), plant architecture and the associated branching patterns play a crucial role in determining crop performance. Singh et al., conducted a study on the introgressive genes from *Sinapis alba* that influence branching traits in *B. juncea*. Their research identified five novel SSR markers and 35 candidate genes linked to enhanced primary and secondary branching, leading to potential improvements in yield performance. This study underscores the importance of genetic resource utilization from related species for crop enhancement.

Basso et al. conducted a genome-wide transcript expression analysis to reveal major genes associated with plant branching in chickpeas (*Cicer arietinum* L.) and lentils (*Lens culinaris* Medik.). By focusing on these vital traits, their research supports the genetic manipulation of branching characteristics, leading to improved light capture and potential yield increases.

Research by Kiros et al. demonstrated that independent genetic factors influence floret and spikelet numbers in *Triticum turgidum* ssp. These traits are integral to maximizing reproductive success and thus yield, opening new avenues for selection and genetic modification.

Jiang et al. identified that a sub-okra leaf shape conferred via chromosomal introgression from *Gossypium barbadense* into *Gossypium hirsutum* can enhance photosynthetic productivity in short-season cotton. This study emphasizes the potential for integrating beneficial traits from related species to improve photosynthetic efficiency and adapt plant architecture to localized growing conditions.

Dai et al. showcased the utility of genome-wide association analysis to uncover candidate genes and haplotypes related to root weight in cucumber (*Cucumis sativus* L.). The implications of root architecture on nutrient uptake and drought resilience are profound, suggesting that root trait optimization is equally essential as above-ground characteristics. The study identified five candidate genes associated with root weight, offering insights into the genetic control of root architecture in cucumber. These findings underscore the significance of roots in breeding programs.

Han et al. mapped dynamic QTL affecting plant height in a RIL population of foxtail millet (*Setaria italica* L.). Their findings contribute valuable insights into the genetic regulation of height, which can influence plant density and light interception strategies, important for optimizing yield.

In conclusion, the research compiled in this Research Topic offers insights into the genetic mechanisms that shape plant architecture in crops, emphasizing their potential to enhance yields and resilience in the face of climate change. Through the application of genomic advancements, research teams have uncovered the genetic foundations that govern key traits related to yield and resilience. The studies featured in this Research Topic encompass a diverse array of crops, elucidating the genetic underpinnings of traits that are critical to agriculture. As future research combines genetic knowledge with advancements in biotechnology, the possibility of creating crop varieties that can withstand stress while optimizing yield through enhanced architecture is becoming more achievable. To move forward with this goal, it will be crucial to encourage interdisciplinary collaboration, establish robust breeding programs, and focus more on the agricultural impact of these findings.

